# Patient-Reported Questionnaires to Identify Adverse Drug Reactions: A Systematic Review

**DOI:** 10.3390/ijerph182211877

**Published:** 2021-11-12

**Authors:** Renly Lim, Lisa Kalisch Ellett, Elizabeth E. Roughead, Phaik Yeong Cheah, Nashwa Masnoon

**Affiliations:** 1 Quality Use of Medicines and Pharmacy Research Centre, UniSA Clinical and Health Sciences, University of South Australia, Adelaide, SA 5000, Australia; lisa.kalisch@unisa.edu.au (L.K.E.); libby.roughead@unisa.edu.au (E.E.R.); 2 Centre for Tropical Medicine and Global Health, Nuffield Department of Medicine, University of Oxford, Oxford OX3 7FZ, UK; phaikyeong@tropmedres.ac; 3 Mahidol-Oxford Tropical Medicine Research Unit, Faculty of Tropical Medicine, Mahidol University, Bangkok 10400, Thailand; 4 The Ethox Centre, Nuffield Department of Population Health, University of Oxford, Oxford OX3 7FZ, UK; 5 Laboratory of Ageing and Pharmacology, Kolling Institute, University of Sydney, St Leonards, NSW 2064, Australia; Nashwa.masnoon@mymail.unisa.edu.au; 6 Department of Pharmacy, Royal North Shore Hospital, St Leonards, NSW 2065, Australia

**Keywords:** adverse drug reactions, adverse events, medication safety, patient safety, questionnaire, side-effects, validity and reliability

## Abstract

Background: This systematic review aims to summarise available patient-reported questionnaires to detect adverse drug reactions (ADRs) that can be utilised by healthcare professionals in clinical practice and to summarise the psychometric properties (validity, reliability, and responsiveness) of the questionnaires. Methods: A systematic literature search was conducted using Medline, Pubmed, Embase, and Emcare databases to screen for articles published between January 2000 and July 2020. Data items regarding validity, reliability, and responsiveness were extracted independently by two authors. The methodological quality was assessed using the COSMIN (Consensus-Based Standards for the Selection of Health Measurement Instruments) checklist. Results: A total of 1563 unique article titles were identified after removing duplicates. Following shortlisting of relevant articles, 19 patient-reported ADR questionnaires were identified. Questionnaires most commonly focused on mental health medications (42.1%, *n* = 8), followed by general questionnaires applicable to any medication (21.1%, *n* = 4). Many questionnaires did not report assessing the validity and reliability of the measurement tool. For example, only 11 questionnaires (58%) mentioned assessing content validity, in addition to criterion or construct testing. Conclusion: This systematic review summarised the available patient-reported questionnaires that can be used in research and clinical practice to identify ADRs. Results of this systematic review highlight the need for more robust validity and reliability testing when developing patient-reported ADR questionnaires.

## 1. Introduction

It has been estimated that the global costs of medication-related harms exceed 40 billion USD annually [1], with half of the harm considered preventable [2]. Adverse drug reactions (ADRs) are unintended and unexpected effects caused by administration of a medication [3]. A 2021 systematic review which included 33 studies estimated that between 8% and 20% persons receiving care in the primary care setting experience ADRs [4]. In Australia, approximately one in five people are likely to be suffering from an ADR at the time they receive a home medicine review [2], which is a government-funded service in Australia where a pharmacist visits the patient in the home to undertake a thorough review of all medications [5].

At the core of preventing or reducing the burden of ADRs is good communication between patients and healthcare professionals regarding any medication-related side-effects being experienced. However, previous studies have shown that patients do not report the adverse reactions that were potentially due to a medication to their doctors, or that there is a mismatch between information provided by the healthcare professionals and information wanted by the patients [6,7]. From a patient’s perspective, the type and severity of ADRs are considered more important than the degree of benefit from the treatment [8]. If the ADRs are not addressed by the healthcare professionals, patients end up lacking knowledge or understanding of the side-effects, do not report the ADRs, and may stop using the medicines [8]. Barriers to patients reporting ADRs to healthcare professionals or regulatory bodies include busy clinical settings, lack of a relationship between patients and practitioners, and issues with pharmacovigilance systems such as lengthy and complicated reporting forms [9,10,11,12]. When ADRs are not detected and not managed properly, patients are likely to stop taking the medications altogether [13].

Patient-reported side-effect questionnaires can be useful to identify ADRs and can contribute to an increased reporting of A26DRs [7,14]. A number of patient-reported side-effect questionnaires such as the Maudsley Side-Effects (MSE) measure [15] and Patient-Reported Adverse Drug Event Questionnaire (PROMISE) [16] have been developed for patients to report side-effects of medications to researchers and healthcare professionals, but the questionnaires are not routinely used in clinical practice. A summary of characteristics and the validity and reliability of existing patient-reported questionnaires will be useful for healthcare professionals to determine the most suitable questionnaire to identify medication harms in practice. Thus, the aims of this systematic review were (i) to identify published patient-reported side-effect questionnaires that can be utilised by healthcare professionals in clinical practice, and (ii) to summarise the psychometric properties (validity, reliability, and responsiveness) of the questionnaires.

## 2. Materials and Methods

The reporting of this systematic review conforms to the Preferred Reporting Items for Systematic Reviews and Meta-Analyses (PRISMA) 2020 statement [17,18]. The PROSPERO International Prospective Register of Systematic Reviews registration number for this systematic review is CRD42020198412.

### 2.1. Search Strategy and Study Selection

The Medline, Pubmed, Embase, and Emcare databases were searched for articles published between January 2000 and July 2020, in humans and in the English language. Databases and search terms were selected by the study investigators, in consultation with an academic librarian specialising in health-related database searches. The search strategy (Supplement File 1) included three main components: “adverse drug reaction”, “patient-report”, and “questionnaire”. Two independent authors (R.L. and N.M.) screened all titles and abstracts using the Covidence software [19]. A third reviewer (L.K.E.) was consulted when there was disagreement about the inclusion of a study. Disagreement was resolved by consensus among the three reviewers. Reference lists of shortlisted studies were screened to identify other relevant articles. If articles referred to existing questionnaires but were not the original article describing the development of the questionnaire, the original article describing the questionnaire was retrieved and included in the review.

Studies were included if they developed general questionnaires which were applicable to medications used for any medical condition or questionnaires targeting medications for common conditions, as guided by a list of common chronic conditions which include cardiovascular disease, chronic obstructive pulmonary disease, asthma, diabetes, arthritis, back pain, cancer, chronic kidney disease, mental health conditions, and osteoporosis [20,21]. Studies focusing on medications used in rare medical conditions were excluded. Studies were also excluded if they translated existing questionnaires in English to other languages; the study describing the original (English) version of the questionnaire was included instead. Questionnaires were excluded if they were for proxy reporting (i.e., not self-reported). Another exclusion criterion was questionnaires focusing only on specific side-effects of medications, such as questionnaires developed to assess peripheral neuropathy from chemotherapy or to assess extrapyramidal side-effects of antipsychotic medications.

### 2.2. Data Extraction

Data extraction items were discussed and agreed upon by two authors (R.L. and N.M.). Data items regarding questionnaire characteristics included (i) the questionnaire name, (ii) author, year, and participant demographics for the primary study developing the questionnaire, (iii) whether questionnaires were general or focused on specific medication classes, (iv) number of items and domains, (v) use of a scoring system, (vi) whether patients can nominate bothersome side-effects, (vii) presence of open-ended questions, and (viii) whether patients were asked if they thought their symptoms were likely medication-related.

Data items regarding validity, reliability, and responsiveness were extracted independently by two authors (R.L. and N.M.) [14,22]. Data items for acceptable development and validity included (i) whether a literature review was undertaken during questionnaire development, (ii) whether a Delphi process or an expert panel was used, (iii) whether the development included patient input, and (iv) criterion validity (comparative testing of a new questionnaire against an established ‘gold-standard’ questionnaire) or construct validity (testing whether the questionnaire assesses the skills and abilities it intends to) [23]. Reliability was assessed as internal consistency (whether the different items in the questionnaire measure the overall general purpose) and test–retest reliability (ability of the questionnaire to produce consistent and very similar results when applied to the same person repeatedly) [23,24]. Responsiveness was measured as the ability of the questionnaire to detect change over time [23,25].

### 2.3. Assessment of Methodological Quality

The methodological quality of all included studies was assessed by one author (R.L.) using the COSMIN (Consensus-Based Standards for the Selection of Health Measurement Instruments) risk of bias checklist (Table S1) [26]. The COSMIN checklist consists of 10 measure properties: PROM development, content validity, structural validity, internal consistency, cross-cultural validity, reliability, measurement error, criterion validity, hypothesis testing for construct validity, and responsiveness. Each measurement property was assessed using a four-point scale: “very good”, “adequate”, “doubtful”, and “inadequate” [26]. The overall rating of the quality was determined using the lowest rating under each measure property [26].

## 3. Results

**Figure 1:**
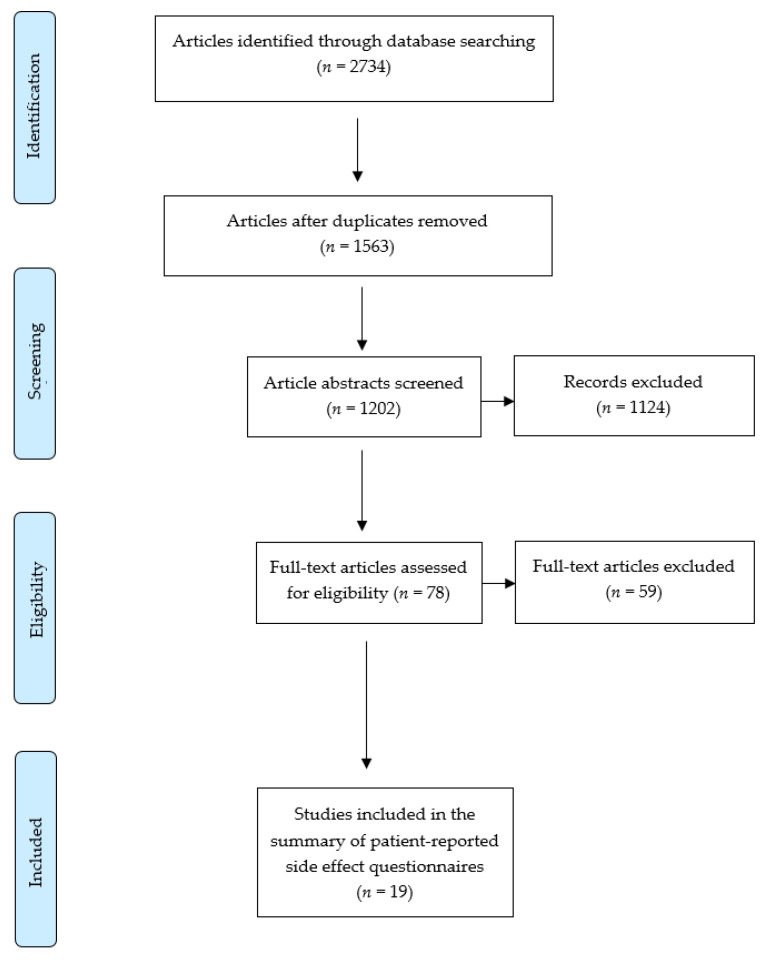
PRISMA flowchart for study selection.

The literature search of the electronic databases identified 2734 studies. After screening 1563 studies once 1171 duplicates were removed, 78 full-text articles were assessed for eligibility. A total of 19 patient-reported questionnaires met the inclusion criteria for this systematic review [7,15,16,27,28,29,30,31,32,33,34,35,36,37,38,39,40,41,42]. The PRISMA flowchart for study selection is presented in Figure 1. The risk of bias assessment using the COSMIN checklist is summarised in Table S1.

Out of the 19 questionnaires, eight (42%) focused on mental health medications [15,27,31,35,36,39,41,42], four (21%) were general questionnaires applicable to any medication [7,16,28,38], two (11%) focused on antiepileptics [30,40], two (11%) focused on inhaled medications for asthma and COPD [29,33], one (5%) focused on diabetes medications [34], one (5%) focused on chemotherapy [37], and one (5%) focused on triptans [32] (Table 1). Table 2 shows a breakdown of the different characteristics of each of the questionnaires such as which medication classes they focus on and the number of side-effect-related items and domains or sections within the questionnaire.

Only nine questionnaires (47%) used a scoring system, with six out of the nine questionnaires having a scoring system with a minimum and a maximum score. Out of these questionnaires mentioning the range of potential scores, the GASS questionnaire was the only tool to provide explicit advice regarding interpretation of scores.

Out of the 19 questionnaires, 11 (58%) included open-ended questions allowing patients to mention any other potential side-effects not explicitly covered by the questionnaire. Six (32%) questionnaires asked patients whether potential side-effects or symptoms were likely medication-related. Six (32%) questionnaires allowed the patients to specifically identify which side-effects were bothersome to them.

Questionnaires differed in their level of detail regarding side effects. For example, the TSQM has a list of 14 questions with five questions relating to side-effects, such as “As a result of taking this medication, do you currently experience any side-effects at all?” and “To what extent do the side-effects interfere with your physical health and ability to function (i.e., strength, energy levels, etc.)?”. Questionnaires such as the SRA had an extensive list of side-effects for patients to indicate whether they have experienced the side-effects or not.

Table 3 shows the results of the validity, reliability, and responsiveness of each questionnaire. With regard to development, 12 (63%) questionnaires explicitly mentioned undertaking a literature review when developing the tool. Eleven (58%) studies mentioned including expert input for the content validity, while 15 (79%) questionnaires involved patients in their development. Criterion or construct testing was undertaken for 11 (58%) of the questionnaires. Out of these questionnaires with criterion or construct testing, four (36%) questionnaires undertook criterion testing by comparing the new questionnaire to a previously published questionnaire, and seven (64%) undertook construct validity testing by employing methods such as exploratory factor analysis using eigen values and accounting for the percentage of total variance. With regard to reliability testing, eight (42%) questionnaires were tested for internal consistency, measured using Cronbach’s alpha. Another aspect of reliability testing is test–retest reliability, which was tested in seven (37%) questionnaires using intraclass correlation coefficients, Cohen’s kappa statistic, and Pearson’s correlation testing. With regard to testing the responsiveness of questionnaires, three (16%) questionnaires assessed the ability of the questionnaires to measure change over time.

## 4. Discussion

When developing questionnaires to identify medication-related side-effects, it is important to consider whether the questionnaire will be general (i.e., focusing on all medications) or specific (i.e., focusing on specific medication classes), involving both patients and health professionals in their development, the presence of scoring systems to quantify the burden of ADRs, and use of open-ended questions to elicit additional information [14,26]. Previous literature has also outlined the importance of considering the validity, reliability, and responsiveness of patient-reported questionnaires when developing and using these tools in clinical practice [14,22]. In this systematic review, we found that different types of patient self-reported questionnaires to identify medication side-effects have been developed, with questionnaires most commonly focusing on identifying side-effects due to mental health medications. There were fewer medication side-effect questionnaires applicable to any medication. The majority of questionnaires (*n* = 15/19) involved patients in their development.

Questionnaires to identify medication side-effects need to be thorough enough to detect the range of ADRs patients experience, as well as provide the opportunity to identify other ADRs which have been missed [14,43]. The challenge in designing patient-reported ADR questionnaires lies in ensuring a comprehensive list of questions to obtain necessary ADR-related information and being practical enough for patients to complete prior to their appointment with their clinician, as well as discussion with their clinician in busy practice settings [44]. We found that the level of detail was variable between the questionnaires; general questionnaires simply asked whether patients had experienced side-effects, whereas condition- or medication-specific questionnaires provided a thorough list of potential ADRs. Questionnaires containing a list of potential ADRs have the advantage of asking about side-effects that patients may not think of; however, the disadvantage is that only known ADRs are listed. Whilst the use of open-ended questions allows patients to mention ADRs which may not already be listed or which they have missed due to not understanding the particular language used, only around 50% of questionnaires included in this systematic review included open-ended questions which allow free-text responses. The lack of time to discuss ADRs during busy practice settings has been identified as a barrier to reporting ADRs [9,10,11]; thus, future patient-reported questionnaires could consider asking patients to list additional side-effects by asking an open-ended question. Open-ended questions do increase response burden and time to complete the survey; therefore, it will be important to assess the duration of time required to complete the questionnaire to reflect the practicality of use.

Previous studies have shown that patients and clinicians may place emphasis on different aspects of treatment [6,8]. The choice to continue medications may depend on the type and severity of side-effects for patients, whereas clinicians’ decision to continue medications may be driven by mortality and morbidity benefits [8]. In this systematic review, we found that less than half of the questionnaires explicitly asked patients to indicate whether the side-effects are bothersome. Considering that the severity of side-effects has an effect on patients’ decision to continue a treatment, it would be important for patients to be able to indicate how severe or bothersome the side-effects actually are for the patients. Additionally, less than half of the questionnaires asked the patients whether the symptom or side-effect is likely medication-related. Uncertainty by patients in whether side-effects are medication-related or not has been identified as a barrier to patients discussing them during appointments [9,10,11,14]. Other barriers to reporting of side-effects are low awareness of patients and healthcare professionals regarding side-effect reporting systems, as well as lack of integration of these questionnaires with electronic health record systems.

The validity of questionnaires is a critical aspect of questionnaire development; however, many questionnaires included in this systematic review did not report assessing the different components of validity. Only around 50% of the questionnaires mentioned assessing content validity, as well as criterion or construct testing. Previous studies have stated the importance of firstly assessing content validity prior to testing other aspects such as construct validity, reliability, and responsiveness [44,45]. Approximately 80% of questionnaires had patient input in their development or validation; it is recommended that all patient-reported questionnaires consider having end-user input to ensure the questionnaires resonate with the target audience [14,26,46].

In terms of reliability of questionnaires, less than 50% of the questionnaires included in this review were tested for internal consistency and test–retest reliability, which fall under the broad category of reliability. A previous literature review of patient-reported side-effect questionnaires undertaken in 2008 argued that it is unclear if internal consistency is a good measure for side-effect questionnaires [14]. This is because there may be a wide range of side-effects which may be unrelated to each other, lowering the internal consistency of the questionnaire, whereas the questionnaire may in fact be appropriate in measuring the different types of potential side-effects [14]. Of the questionnaires included in our systematic review that were tested for internal consistency, the high Cronbach’s α of more than 0.7 for all the questionnaires tested suggests that internal consistency can be used a measure of reliability for patient side-effect questionnaires.

Factors that can affect choice of questionnaire for use in research or practice include the population in which the tool will be used, the time taken to complete the tool, psychometric properties of the questionnaires, and presence of a scoring system for practicality reasons. Findings of our systematic review suggest that the Glasgow Antipsychotic Side-Effect Scale (GASS) [41], Patient Assessment Questionnaire (PAQ) [36], and Maudsley Side-Effects (MSE) [15], which have adequate psychometric properties and a scoring system, are suitable for use in clinical practice to identify side-effects of antipsychotic medications. The Patient-Reported Adverse Drug Event Questionnaire [16], a general questionnaire which consists of a comprehensive list of adverse drug events and has good psychometric properties, is considered suitable for use in clinical practice.

To the best of our knowledge, this is the first systematic review that summarised the range of patient-reported questionnaires to identify side-effects of medications. Previous systematic reviews focused on specific types of medications, such as the 2015 systematic review which included rating scales to measure the side-effects of antipsychotic medications [47]. A limitation of this systematic review is the exclusion of non-English studies which may have subsequently removed questionnaires in other languages. The focus of our systematic review was to identify questionnaires that can be used in clinical practice to detect side-effects of medications used for any condition or common chronic conditions. As a result, there may be questionnaires for rare conditions which employ novel ways of obtaining side-effect-related information that were excluded in our systematic review. While we used a comprehensive search strategy and covered several databases, we may not have identified all questionnaires available that meet our inclusion criteria. There may be a range of terms which studies may use to refer to the concept of “patient report” such as “patient perception” which may have resulted in some questionnaires being missed.

## 5. Conclusions

This systematic review summarised the available patient-reported questionnaires that can be used in research and clinical practice to identify ADRs. Questionnaires that have been developed to date most commonly focused on identifying side-effects due to mental health medications. Patient-reported side-effect questionnaires can be a useful tool to identify ADRs that may not otherwise be reported by patients, potentially facilitating improved medication adherence and patient outcomes. Results of this systematic review highlight the need for more robust validity and reliability testing when developing patient-reported ADR questionnaires. Future studies could consider assessing the applicability and effectiveness of these tools in clinical practice to improve patient care and outcomes.

## Figures and Tables

**Table 1:** List of questionnaires included in this systematic review.

General vs. Focusing on Specific Medications or Disease/Health Conditions	Name of Questionnaire
Mental health medications, *n* = 8 [15,27,31,35,36,39,41,42]	My Medicines and Me (M3Q) Approaches to Schizophrenia Communication Self-Report Checklist (ASC-SR) Subjects’ Response to Antipsychotics (SRA) Glasgow Antipsychotic Side-Effect Scale (GASS) Patient Assessment Questionnaire (PAQ) Systematic Monitoring of Adverse Events Related to Treatments (SMARTS) Maudsley Side-Effects (MSE) measure Antidepressant Side-Effect Checklist (ASEC)
General questionnaires, *n* = 4 [7,16,28,38]	Generic symptoms questionnaire Treatment Satisfaction Questionnaire for Medication (TSQM) Patient-Reported Adverse Drug Event Questionnaire Patient-Reported Outcome Measure Inquiry into Side-Effects (PROMISE)
Antiepileptics, *n* = 2 [30,40]	Side-Effect Checklist Assessment of Side-Effects in AED treatment (SIDAED)
Asthma and chronic obstructive pulmonary disease, *n* = 2 [29,33]	Satisfaction with Asthma Treatment Questionnaire Inhaled Corticosteroids Questionnaire (ICQ)
Diabetes, *n* = 1 [34]	Patient’s Qualitative Assessment of Treatment (PQAT)
Chemotherapy, *n* = 1 [37]	Common Terminology Criteria for Adverse Events Side-Effects Questionnaire
Triptans, *n* = 1 [32]	Triptans Questionnaire

**Table 2:** Characteristics of patient-reported questionnaires to detect ADRs.

Questionnaire Name, Primary Author, Year, and Country	Participant Demographics for Development	General vs. Focusing on Specific Medications or Disease/Health Conditions	Number of Side-Effect-Related Items	Domains	Response Categories	Scoring	Patients Nominate Bothersome Side-Effects	Open-Ended Questions	Asking Whether Side-Effects Likely Medication-Related
Generic Symptoms Questionnaire Jarernsiripornkul, 2001, UK [7]	837 patients, mean age 50.5, 34% male	General	22	18 categories of body systems	Yes/no or ticking checkbox of potential side-effects	None	Yes	Yes	Yes
Treatment Satisfaction Questionnaire for Medication (TSQM) Atkinson 2004, USA [28]	567 patients, mean age 50.5 years	General	5	4 domains: effectiveness, side-effects, treatment satisfaction, and convenience	7-point scale ranging from ‘extremely satisfied’ to ‘extremely dissatisfied’	0–100	No	None	Yes
Patient-Reported Adverse Drug Event Questionnaire Vries, 2013, Netherlands [16]	135 patients, mean age 65 years, 60% male	General	252 ADEs categorised in body categories	16 categories of body systems	Yes/no	None	Yes	Yes	Yes
Patient-Reported Outcome Measure, Inquiry into Side-Effects (PROMISE) Schoenmakers, 2017, Netherlands [38]	180 patients, mean age 73 years, 48% male	General	One item with 22 symptoms	5 domains: health status, beliefs and concerns about medicines, self-efficacy in understanding and using medicines, medication adherence, and potentially drug-related symptoms	Yes/no	None	No	Yes	Yes
My Medicines and Me (M3Q) Ashoorian 2015, Australia [27]	78 (10 patients, 8 carers, 28 physicians, 10 nurses, and 22 pharmacists), age range 21–80, 40% male	Mental health	32	3 domains: current medications, side-effects, and general questions	Yes/no	None	Yes	Yes	Yes
Approaches to Schizophrenia Communication Self-Report Checklist (ASC-SR) Dott 2001, US, Canada, and UK [31]	152 patients and 21 psychiatrists and case workers	Antipsychotics	17	1 domain: side-effects	‘I have had this experience recently’ and ‘I would like to talk about this to a nurse of doctor’	None	Yes	Yes	No
Subjects’ Response to Antipsychotics (SRA) Wolters 2006, Netherlands [42]	320 patients, mean age 35 years, 73% male	Antipsychotics	74	9 domains: weight gain, sexual anhedonia, sedation, affective flattening, extrapyramidal symptoms, diminished sociability, increased sleep, recovery, and other	‘No’, ‘yes to a certain degree’, and ‘yes to a high degree’	Range of total scores not explicitly mentioned	No	None	No
Glasgow Antipsychotic Side-Effect Scale (GASS) Waddell, 2008, UK [41]	50 patients, age range 18–65 years, 47% male	Second generation antipsychotics	22	9 domains: sedation and central nervous system (CNS), cardiovascular, extrapyramidal, anticholinergic, gastrointestinal, genitourinary, screening for diabetes mellitus, prolactinaemia, and weight gain	Q1–30 scored 0 (never) to 3 (everyday), Q21–22 scored 0 for no and 3 for yes.	Divided into 3 sections; 0–21 = absent/mild side-effects; 22–42 = moderate side-effects, and 43–63 = severe side-effects.	Yes	None	No
Patient Assessment Questionnaire (PAQ) Mojtabai 2012, US [36]	300 patients, mean age 46.7 years, 57% male	Antipsychotics	40	5 domains: general distress, side-effects, psychotic symptoms, cognitive symptoms, and sleep	0 = ‘not at all’ to 4 = ‘extremely/very much’	Maximum total score of 160	No	None	No
Systematic Monitoring of Adverse Events Related to Treatments (SMARTS) Haddad, 2014, UK [35]	Not specified	Antipsychotic	12	9 domains: extrapyramidal symptoms, sexual dysfunction, hyperprolactinaemia, postural hypotension, sedation, appetite and weight change, gastrointestinal side-effects, urinary symptoms, and affective side-effects	Yes/no	None	Yes	Yes	No
Maudsley Side-Effects (MSE) measure Wykes 2017, UK, USA, and Spain [15]	108 (patients, psychiatrists and pharmacists), mean age 44.2 years, 46.3% male	Antipsychotics	53	2 domains: demographics and side-effects	Rating from ‘not at all’ to ‘severe’	Total side-effects (0–53), total intensity (0–159), total distress (0–53), and total life impact (0–159)	Yes	Yes	No
Antidepressant Side-Effect Checklist (ASEC) Uher 2009, Europe [39]	811 patients, mean age 42.5 years, 36.6% male	Antidepressants, SSRI (escitalopram) and TCA (nortriptyline)	21	3 domains: potential side-effects, any treatment for side-effects, and if side-effects led to antidepressant discontinuation	4-point scale ranging from 0 = absent to 3 = severe	Range of total scores not explicitly mentioned	No	Yes	Yes
Side-Effect Checklist Carpay 2005, Netherlands [30]	346 patients, mean age 51.9 years, 50.4% male	Antiepileptics	30	8 domains: general CNS, motor problems, gastrointestinal complaints, cognition, visual, mood, behaviour, cosmetic, and sleep problems	Side-effects dichotomised as present or not present, side-effect severity rating: 4-point scale ranging from ‘none’ to ‘very severe’	Range of total scores not explicitly mentioned	No	None	No
Assessment of Side-Effects in AED Treatment (SIDAED) Uijl 2006, Netherlands [40]	173 patients, mean age 48 years, 50% male	Antiepileptics	46	10 domains: general CNS, behaviour, depressive symptoms, cognitive function, motor problems/coordination, visual complaints, headache, cosmetic and dermatological complaints, gastrointestinal complaints, and sexuality and menses	Severity rating from 0 = ‘no problem’ to 3 = ‘serious problem’, duration of complaints also scored (for example, since a few weeks vs. since months)	0–138	No	None	No
Satisfaction with Asthma Treatment Questionnaire Campbell, 2003, UK [29]	131 patients, mean age 45, 34% male	Inhaled asthma medicines	26	4 domains: effectiveness of treatment, ease of use, medication burden, and side-effects and worries	1 (strongly disagree) to 7 (strongly agree)	None	No	None	No
Inhaled Corticosteroids Questionnaire (ICQ) Foster 2006, Netherlands and Scotland [33]	395 patients, mean age 50 years, 47% male	Inhaled corticosteroids	57	8 domains: voice, cough, oropharynx, taste, mouth, skin, mood, and other	7-point scale ranging from 0 = ‘not at all’ to 6 = ‘a very great deal’	Out of 100	No	Yes	No
Patient’s Qualitative Assessment of Treatment (PQAT) Gater, 2020, UK [34]	57 patients, mean age 57, 58% male	Type 1 and 2 diabetes	4	4 domains: benefits of the drug, disadvantages of the drug, willingness to continue with the drug, and balance between benefits and disadvantages	Combination of qualitative answers, yes/no and scales of 0–10 and −3 to 3	Range of total scores not explicitly mentioned	No	Yes	No
Common Terminology Criteria for Adverse Events Side-Effects Questionnaire Pearce, 2017, Australia [37]	441 patients, majority (59.7%) in the 45–65 years group, 26.1% male	Chemotherapy	9	9 domains: diarrhoea, vomiting, chest pain or angina, constipation, dyspnoea, fatigue, mucositis, pain, and rash	0 = not present to 5 = severe	None	No	None	No
Triptans Questionnaire Feleppa, 2004, Italy [32]	108 patients, mean age 39.5, 13% male	Triptans	2	2 domains: unprompted side-effects and prompted side-effects	Combination of free text, yes/no, rating 1 = mild to 3 = severe	None	No	Yes	No

ADE, adverse drug events; CNS, central nervous system; SSRI, selective serotonin reuptake inhibitors; TCA, tricyclic antidepressants.

**Table 3:** Validity, reliability, and responsiveness of patient-reported side-effect questionnaires to identify adverse drug reactions.

	Validity				Reliability		Responsiveness
Questionnaire Name, Primary Author, Year, and Country	Literature Review	Delphi/Expert Panel (Content Validity)	Patient Input	Criterion or Construct Testing	Internal Consistency	Test–Retest Reliability	Ability to Detect Change over Time
Generic Symptoms Questionnaire Jarernsiripornkul, 2001, UK [7]	Previously published work used as a basis	Unclear	Initially piloted in 11 patients followed by further patient pilot groups	Reporters of musculoskeletal symptoms taking statins had significantly higher mean creatinine kinase level than those not reporting any musculoskeletal symptoms (207.35 ± 155.40 vs. 143.95 ± 83.07 U/L, respectively; *p* = 0.037)	None	None	None
Treatment Satisfaction Questionnaire for Medication (TSQM) Atkinson 2004, USA [28]	Literature review regarding patient satisfaction with medications across various therapeutic areas	Unclear	Three focus group with patients allowing integration of the patients’ perspectives and initial item reduction and scaling	Multistep exploratory factor analyses (EFA) used. First EFA produced three factors (eigenvalue more than 1.7 explaining 75.6% of overall variance); second EFA yielded final instrument (eigenvalue =2.3 explaining 79.1% of total variance)	High Cronbach’s α of around 0.88 for each domain	ICC values were high when comparing results at two timepoints separated by 7–4 days: 0.784 for effectiveness, 0.737 for convenience and 0.759 for t global satisfaction	None
Patient-Reported Adverse Drug Event Questionnaire Vries, 2013, Netherlands [16]	Common Terminology Criteria for Adverse Events version 4.0 and existing symptom and ADE checklists used	Unclear	Cognitive debriefing interviewing with patients to eliminate ambiguity in questions	Construct validity—patients who reported side-effects (*n* = 37) had a lower general quality of life and physical health than those not reporting side-effects (*p* < 0.05). Concurrent validity—in comparison with TSQM, this questionnaire had a sensitivity of 38% and positive predictive value of 79% for assessing side-effects associated with metformin	None	Test–retest reliability was acceptable at patient level (*k* = 0.50, PPA 0.64)	None
Patient Reported Outcome Measure, Inquiry into Side-Effects (PROMISE) Schoenmakers, 2017, Netherlands [38]	Existing instruments and literature regarding side effects of drugs most frequently used in the Netherlands used	Unclear	Pretested in patients eligible for a medication review to assess whether the items were well understood	None	None	None	None
My Medicines and Me (M3Q) Ashoorian 2015, Australia [27]	Previous self-report questionnaires assessing subjective experiences of medication side-effects used	Focus groups with psychiatrists, general practitioners, mental health nurses, and pharmacists	Focus group with carers and mental health patients	Spearman’s nonparametric coefficient of correlation was high and statistically significant (*ρ* = 0.724, *p* < 0.001)	High Cronbach’s α of 0.929	None	None
Approaches to Schizophrenia Communication Self-Report Checklist (ASC-SR) Dott 2001, US, Canada and UK [31]	Item generation through literature search	Steering group consisted of psychiatrists	Patient input regarding usefulness of checklist	None	None	None	None
Subjects’ Response to Antipsychotics (SRA) Wolters 2006, Netherlands [42]	Unclear	Clinical experts categorised items into subscales	Semi-structured interviews with 77 patients for item generation	Moderate to low correlations between SRA and Subjective Wellbeing on Neuroleptics (SWN) subscales	Cronbach’s α of the subscales were between 0.69 and 0.93	Pearson’s *r* correlation between scores tested 1 week apart was 0.76 for all but two subscales (sexual anhedonia and affective flattening)	None
Glasgow Antipsychotic Side-Effect Scale (GASS) Waddell, 2008, UK [41]	Existing questionnaires and information from the British National Formulary and the pharmaceutical industry used	Discussion with members of the mental health team	Focus group of patients taking antipsychotics ranked the list of side-effects in terms of acceptability	GASS scores for two groups taking and not taking antipsychotics differed significantly (Mann–Whitney U-test, U = 2336, *p* < 0.0001) with a mean of 14.3 for those on antipsychotics and 3.6 for those not on antipsychotics	None	Good test–retest reliability, with κ = 0.72.	None
Patient Assessment Questionnaire (PAQ) Mojtabai 2012, US [36]	Unclear	Experts in psychiatry, social and behavioural sciences, and psychometrics used	Patient focus groups leading to questionnaire refinement	Exploratory factor analysis and visual inspection of scree plots identified five factors with eigenvalues more than 1 (accounting for 50.4% of the variance)	Cronbach’s α of 0.85 for the side effect subscale	None	None
Systematic Monitoring of Adverse Events Related to Treatments (SMARTS) Haddad, 2014, UK [35]	11 side-effects included after literature search	Developed over a series of group meetings by an international faculty of 12 experts (including psychiatrists, a general physician, and psychopharmacologist)	No	None	None	None	None
Maudsley Side-Effects (MSE) measure Wykes 2017, UK, USA and Spain [15]	Published literature of antipsychotic side-effect rating scales used	Delphi exercise with psychiatrists and pharmacists	Patient focus groups used	Compared to the GASS tool, the MSE and GASS subscales were highly correlated (total side-effects: Pearson’s correlation, *r* = 0.8, intensity: *r* = 0.8, and distress: *r* = 0.7, *p* < 0.001 in all cases)	Cronbach’s α for the total side-effects score was 0.96	Scores were highly correlated (0.81–0.96) between 6 and 8 days, with no statistically significant differences in the mean scores	None
Antidepressant Side-Effect Checklist (ASEC) Uher 2009, Europe [39]	List of adverse effects compiled from the literature	Scientists, clinicians, and industrial partners involved	No	Agreement between the self-rated ASEC and interviewer-rated UKU was good, with kappa ranging from 0.55 for insomnia to 0.89 for dry mouth	Average inter-item covariance was 0.05 and Cronbach’s α was 0.78	None	Dryness of mouth was significantly more frequent during treatment with escitalopram (OR = 1.46) and nortriptyline (OR = 9.04) compared to antidepressant-free baseline
Side-Effect Checklist Carpay 2005, Netherlands [30]	Unclear	Unclear	Community-based patients completed the checklist	None	None	None	None
Assessment of Side-Effects in AED Treatment (SIDAED) Uijl 2006, Netherlands [40]	Unclear	Unclear	Patients asked to complete questionnaire	None	None	None	In a trial involving 111 adults with epilepsy randomised to either intervention (adjustment of antiepileptics based on SIDAED responses) or control (treatment unchanged) over 7 months, there was a decrease in complaints by intervention, rate ratio of 1.34 (not statistically significant)
Satisfaction with Asthma Treatment Questionnaire Campbell, 2003, UK [29]	Preliminary instrument based on literature review	Preliminary instrument based on expert opinion, focus groups, and literature findings	Two focus groups to understand patient perception of asthma treatment regimens and problems	Eight items showed factor loadings of <0.35 on any factor or had high factor loadings on more than one factor and were excluded	Cronbach’s α ranged from 0.71–0.88	Test/retest reliability (intra correlation coefficients) ranged from 0.66–0.74	None
Inhaled Corticosteroids Questionnaire (ICQ) Foster 2006, Netherlands and Scotland [33]	Unclear	Expert panel reviewed side-effect items	In-depth interviews and focus groups with patients to talk about their experiences of ICS side-effects	All three construct validity hypotheses were well supported: (i) statistically significant difference existed in scores for 14 domains with the high ICS dose group scoring highest; (ii) ICS dose independently predicted ICQ scoring after adjusting for confounders; (iii) greater convergence existed between local ICQ domains than between local and systemic domains	Excellent internal consistency: Cronbach’s α = 0.98	Test–retest intraclass correlation coefficients were ≥0.69 for all but the ‘facial oedema’ domain	Comparing different dosing regimens of inhaled ciclesonide and fluticasone over 12 or 24 weeks, no significant score changes were observed from baseline
Patient’s Qualitative Assessment of Treatment (PQAT) Gater, 2020, UK [34]	Unclear	Initial items were developed by the experts	Cognitive testing of the initial version was conducted among 7 patients with type 1 and type 2 diabetes	No	None	None	None
Common Terminology Criteria for Adverse Events Side-Effects Questionnaire Pearce, 2017, Australia [37]	The National Cancer Institute Common Toxicity Criteria version 4 was adapted in English	Unclear	No	None	None	None	None
Triptans Questionnaire Feleppa, 2004, Italy [32]	Unclear	Unclear	No	None	None	None	None

ASEC, Antidepressant Side-Effect Checklist; EFA, exploratory factor analyses; GASS, Glasgow Antipsychotic Side-Effect Scale; ICC, intraclass correlation coefficient; ICS, Inhaled Corticosteroids Questionnaire; *k*, Cohen’s kappa coefficient; MSE Maudsley Side-Effects; *r*, Pearson correlation coefficient; SIDAED, Assessment of Side-Effects in AED Treatment; *ρ*, Spearman’s rank correlation coefficient; SRA, Subjects’ Response to Antipsychotics; TSQM, Treatment Satisfaction Questionnaire for Medication; UKU, Udvalg for Kliniske Undersogelser.

## Data Availability

No new data were created or analyzed in this study. Data sharing is not applicable to this article.

## Supplementary Materials

The following are available online at https://www.mdpi.com/1660-4601/18/22/11877/s1: Supplement File 1. Search strategy; Table S1. COSMIN (Consensus-Based Standards for the Selection of Health Measurement Instruments) risk of bias checklist.
