# Correction: Lim et al. Patient-Reported Questionnaires to Identify Adverse Drug Reactions: A Systematic Review. *Int. J. Environ. Res. Public Health* 2021, *18*, 11877

**DOI:** 10.3390/ijerph191811209

**Published:** 2022-09-07

**Authors:** Renly Lim, Lisa Kalisch Ellett, Elizabeth E. Roughead, Phaik Yeong Cheah, Nashwa Masnoon

**Affiliations:** 1 Quality Use of Medicines and Pharmacy Research Centre, UniSA Clinical and Health Sciences, University of South Australia, Adelaide, SA 5000, Australia; 2 Centre for Tropical Medicine and Global Health, Nuffield Department of Medicine, University of Oxford, Oxford OX3 7FZ, UK; 3 Mahidol-Oxford Tropical Medicine Research Unit, Faculty of Tropical Medicine, Mahidol University, Bangkok 10400, Thailand; 4 The Ethox Centre, Nuffield Department of Population Health, University of Oxford, Oxford OX3 7FZ, UK; 5 Laboratory of Ageing and Pharmacology, Kolling Institute, University of Sydney, St Leonards, NSW 2064, Australia; 6 Department of Pharmacy, Royal North Shore Hospital, St Leonards, NSW 2065, Australia

## Text Correction

There was an error in the original publication [1]. We stated that none of the questionnaires included information on how long it took to complete, which is incorrect. The following corrections have been made.

Section 3, Paragraph 3: “None of the questionnaires stated how long it takes to complete each questionnaire” has been removed;

Section 4, Paragraph 1: “None of the questionnaires stated how long it takes to complete each questionnaire, which would affect the practicality of using the questionnaires in day-to-day practice” has been removed; and

Section 4, Paragraph 3: “Although none of the questionnaires stated how long it takes to complete each questionnaire” has been removed.

The authors apologize for any inconvenience caused and state that the scientific conclusions are unaffected. The original publication has also been updated.
