# Supplementary material for: Patient-Reported Questionnaires to Identify Adverse Drug Reactions: A Systematic Review
Source: Int J Environ Res Public Health. 2021 Nov 12;18(22):11877. doi: 10.3390/ijerph182211877 (PMC8624083; doi:10.3390/ijerph182211877)
Supplement: Supplementary file 1 [file ijerph-18-11877-s001.zip › Supplement file 1 .pdf]

Supplement file 1

Search strategy

The following search terms (Medical Subject Headings [MeSH] and keywords) were used:

‘adverse drug reaction/’ (MeSH and keyword) OR ‘adverse medicine reaction*’ (keyword) OR ‘adverse medication reaction*’ (keyword) OR ‘side effect/’ (MeSH and keyword)

AND
‘self report/’ (MeSH and keyword) OR ‘self-report*’ (keyword) OR ‘patient-report*’ (keyword) OR ‘patient report*’ (keyword)
AND
‘questionnaire*’ (keyword) OR ‘checklist*’ (keyword)
The following search terms (MeSH and keywords) were used in MEDLINE:

‘adverse drug reaction*’ (keyword) OR ‘adverse medicine reaction*’ (keyword) OR ‘adverse medication reaction*’ (keyword) OR ‘side effect*’ (keyword) OR ‘Drug-Related Side Effects and Adverse Reactions/’(MeSH)

AND

‘self report/’ (MeSH and keyword) OR ‘self-report*’ (keyword) OR ‘patient-report*’ (keyword) OR ‘patient report*’ (keyword)
AND
‘questionnaire*’ (keyword) OR ‘Surveys and Questionnaires/’ (MeSH) OR ‘checklist/’ (MeSH and keyword)
If articles referred to existing questionnaires but were not the original research article describing the formulation and development of the questionnaire, the original article describing the questionnaire was found and included in the review.
