# Supplementary material for: Patient-Reported Questionnaires to Identify Adverse Drug Reactions: A Systematic Review
Source: Int J Environ Res Public Health. 2021 Nov 12;18(22):11877. doi: 10.3390/ijerph182211877 (PMC8624083; doi:10.3390/ijerph182211877)
Supplement: Supplementary file 1 [file ijerph-18-11877-s001.zip › Table S1.pdf]

Table S1: Methodological quality of studies included

S/No.	PROM	Reference	PROM development	Methodological quality of studies/ quality of measurement properties of PROM								
				Content validity	Structural validity	Internal consistency	Cross-cultural validity	Reliability	Measurement error	Criterion validity	Hypothesis testing	Responsiveness
1	Generic symptoms questionnaire	Jarernsiripornkul 2001	Adequate	Inadequate	N/a	N/a	N/a	N/a	N/a	Inadequate	N/a	N/a
2	TSQM	Atkinson 2004	Doubtful	Inadequate	Adequate	Very good	N/a	Adequate	N/a	N/a	N/a	N/a
3	Patient-reported Adverse Drug Event Questionnaire	Vries 2013	Doubtful	Inadequate	N/a	N/a	N/a	Doubtful	N/a	N/a	Adequate	N/a
4	PROMISE	Schoenmakers 2017	Doubtful	Inadequate	N/a	N/a	N/a	N/a	N/a	N/a	N/a	N/a
5	M3Q	Ashoorian 2015	Adequate	Inadequate	N/a	Very good	N/a	N/a	N/a	N/a	N/a	N/a
6	ASC-SR	Dott 2001	Doubtful	Inadequate	N/a	N/a	N/a	N/a	N/a	N/a	N/a	N/a
7	SRA	Wolters 2006	Adequate	Inadequate	N/a	Very good	N/a	Adequate	N/a	Very good	Adequate	N/a
8	GASS	Waddell 2008	Adequate	Inadequate	N/a	N/a	N/a	Adequate	N/a	N/a	Very good	N/a
9	PAQ	Mojtabai 2012	Doubtful	Inadequate	Adequate	Very good	N/a	N/a	N/a	N/a	N/a	N/a

10	SMARTS	Haddad 2014	Adequate	Inadequate	N/a	N/a	N/a	N/a	N/a	N/a	N/a	N/a
11	MSE	Wykes 2017	Adequate	Doubtful	N/a	Very good	N/a	Adequate	N/a	Inadequate	N/a	N/a
12	ASEC	Uher 2009	Adequate	Inadequate	N/a	Very good	N/a	N/a	N/a	N/a	Inadequat e	Inadequate
13	Side-effect checklist	Carpay 2005	Inadequate	Inadequate	N/a	N/a	N/a	N/a	N/a	N/a	N/a	N/a
14	SIDAED	Uijl 2006	Inadequate	Inadequate	N/a	N/a	N/a	N/a	N/a	N/a	N/a	Inadequate
15	Satisfaction with Asthma Treatment Questionnaire	Campbell 2003	Doubtful	Doubtful	Doubtful	Very good	N/a	Adequate	N/a	N/a	N/a	N/a
16	ICQ	Foster 2006	Inadequate	Doubtful	N/a	Very good	N/a	Adequate	N/a	N/a	N/a	Inadequate
17	PQAT	Gater 2020	Inadequate	Inadequate	N/a	N/a	N/a	N/a	N/a	N/a	N/a	N/a
18	Common Terminology Criteria for Adverse Events Side Effects Questionnaire	Pearce 2017	Inadequate	Inadequate	N/a	N/a	N/a	N/a	N/a	N/a	N/a	N/a
19	Triptans Questionnaire	Feleppa 2004	Inadequate	Inadequate	N/a	N/a	N/a	N/a	N/a	N/a	N/a	N/a

N/a: Data not available for assessment
